# Los cambios inmunofenotípicos en tiempo real en la leucemia linfoblástica pediátrica de tipo B tienen implicaciones para la detección de la enfermedad mínima residual

**DOI:** 10.1515/almed-2025-0104

**Published:** 2025-08-29

**Authors:** Omer Javed, Neelum Mansoor, Naeem Jabbar, Hamza Khan, Talha Israr, Sidra Maqsood, Saba Jamal, Fatima Meraj

**Affiliations:** Servicio de Hematología, Hospital y Red Sanitaria Indus, Karachi, Pakistan; Servicio de citogenética y hematología, Hospital y Red Sanitaria Indus, Karachi, Pakistan; Departamento de Oncología Pediátrica, Hospital y Red Sanitaria Indus, Karachi, Pakistan; ORIC, Hospital y Red Sanitaria Indus, Karachi, Pakistan; Servicio de Patología y Unidad de Transfusiones Sanguíneas, Hospital y Red Sanitaria Indus, Karachi, Pakistan

**Keywords:** leucemia linfoblástica aguda, células blásticas, citometría de flujo, modulación inmunofenotípica, enfermedad mínima residual

## Abstract

**Objetivos:**

El seguimiento de los pacientes con leucemia aguda resulta fundamental para la adecuada estratificación del riesgo y la evaluación de la respuesta a la quimioterapia. El método de seguimiento más fiable es la detección de enfermedad mínima residual (EMR), ya que, a través de ella, se identifican diferencias de expresión de antígenos entre las células leucémicas, los hematógonos y las células B benignas maduras. La modulación de la expresión de antígenos durante el tratamiento con agentes antineoplásicos podría dificultar el análisis y detección de EMR. En el presente estudio investigamos la modulación inmunofenotípica (MI) durante las distintas fases de la quimioterapia en pacientes pediátricos con leucemia linfoblástica aguda (LLA-B), mediante la aplicación de un protocolo de BFM modificado, y evaluamos sus posibles implicaciones a la hora de detectar EMR.

**Métodos:**

El estudio fue llevado a cabo en el Servicio de Hematología del Hospital y Red Sanitaria Indus, en Karachi (Pakistán). Se incluyeron todos los casos con resultado positivo para EMR de LLA-B en pacientes pediátricos (1 mes -16 años de edad) entre abril de 2019 y marzo de 2022. La presencia de EMR se determinó mediante aspirado de médula ósea, utilizando citometría de flujo de ocho colores. Se incluyeron datos de 203 pacientes con resultado positivo de EMR en la médula ósea durante el periodo de evaluación. La MI se determinó realizando un análisis comparativo de los cambios observados en la intensidad media de fluorescencia (IMF) de nueve antígenos clave expresados en las células leucémicas.

**Resultados:**

Se observaron cambios estadísticamente significativos en los niveles de IMF de los antígenos expresados en los blastos leucémicos, así como en las células B benignas maduras. Todas las muestras analizadas revelaron MI en diferentes grados. Los resultados obtenidos confirman la presencia de cambios inmunofenotípicos en CD10, CD19, CD34, CD45, TdT, y CD66 durante la quimioterapia.

**Conclusiones:**

Si bien la determinación de la EMR facilita el seguimiento de la enfermedad, es necesario tener en cuenta y analizar meticulosamente la MI de los diferentes antígenos expresados en los blastos leucémicos, con el fin de evitar resultados erróneos.

## Introducción

La leucemia linfoblástica aguda (LLA) es el tipo más común de cáncer pediátrico, representando entre el 80 y el 85 % de los casos de leucemia linfoblástica aguda de células B (LLA-B) [1]. El inmunofenotipo de las células malignas se suele determinar mediante citometría de flujo [2]. La realización de un seguimiento durante el manejo de la leucemia aguda resulta fundamental a la hora de estratificar el riesgo y evaluar la respuesta a la quimioterapia, siendo el análisis de enfermedad mínima residual (EMR), que ha evolucionado en los últimos años, la estrategia de seguimiento de mayor fiabilidad [2]. La detección de EMR mediante citometría de flujo o reacción en cadena de la polimerasa (PCR) es un importante indicador pronóstico, siendo crucial en la predicción de los resultados clínicos [3]. La determinación de EMR mediante citometría de flujo se basa en el principio de que las células leucémicas presentan patrones inusuales de expresión de antígenos, lo que los distingue de las células precursoras en proceso de maduración, esto es, los hematógonos [4]. Se trata de un método muy extendido que se emplea para evaluar la respuesta de la enfermedad a la terapia, presentando una sensibilidad muy elevada.

La detección de blastos residuales y hematógonos mediante citometría de flujo, sumada a las variaciones en la expresión de diferentes antígenos inducidas por la quimioterapia durante el curso de la enfermedad, han atraído el interés de la comunidad científica internacional. Así, estos marcadores se analizan en diferentes momentos a lo largo del tratamiento para vigilar el estado de remisión [5]. En los pacientes con LLA-B, durante el tratamiento se producen variaciones en la expresión de antígenos en los blastos residuales, especialmente durante la quimioterapia de inducción, lo que puede dificultar el análisis e interpretación de la EMR [6].

Los agentes antineoplásicos modulan la expresión de los diferentes marcadores empleados para determinar el fenotipo diagnóstico y realizar el seguimiento de la EMR. La modulación de la expresión de CD10, CD20, CD34, TdT y CD45 ha quedado demostrada en la literatura, especialmente durante la fase glucocorticoide (fase GC) de la terapia de inducción [7]. Dichos cambios fenotípicos suponen un obstáculo a la hora de evaluar la EMR, ya que pueden llevar a interpretaciones erróneas y derivar en la comunicación de un falso negativo o falso positivo en el informe de resultados.

Si bien existe información muy escasa sobre los patrones de modulación inmunofenotípica en la leucemia aguda en nuestro entorno, en un estudio reciente, se observó una frecuencia relativamente mayor de la presencia de EMR en nuestros pacientes pediátricos con LLA-B [8]. Los resultados del presente estudio demuestran ampliamente la expresión y variación de marcadores inmaduros y de un determinado linaje celular. La realización de un estudio unicéntrico con un mayor tamaño muestral permitiría comparar nuestros datos con los de otros estudio e identificar cambios significativos en la expresión de antígenos, que facilitarían la interpretación de la EMR. En nuestro estudio, analizamos las tendencias en la modulación inmunofenotípica de las células leucémicas y comparamos sus patrones con los de las células B no leucémicas en la misma muestra que pudieran estar también presentes durante las diferentes fases de la quimioterapia.

## Materiales y métodos

### Pacientes

Se llevó a cabo un estudio retrospectivo observacional en el Servicio de Hematología del Hospital y la Red Sanitaria Indus en Karachi (Pakistán), entre abril de 2019 y marzo de 2022. Se incluyó a todos los pacientes con diagnóstico reciente de LLA-B con edades comprendidas entre 1 mes y 16 años. Siguiendo los criterios de estratificiación del riesgo del National Cancer Institute (NCI), se procedió a administrar a los pacientes vincristina, asparaginasa, dexametasona, doxorrubicina/daunorrubicina, 6MP y metotrexato intratecal aplicando el protocolo de quimioterapia Berlin-Frankfurt-Munster (BFM) modificado. Dicho protocolo consiste en una profase de prednisona de una semana de duración a una dosis de 60 mg/m^2^/día por vía oral o intravenosa, seguida de una evaluación de la respuesta el día 8 de la profase en base al porcentaje de blastos en sangre periférica.

### Citometría de flujo

El diagnóstico y la detección de EMR se realizaron mediante citometría de flujo. Se observó modulación inmunofenotípica (MI) en la expresión de antígenos de TdT, CD34, CD10, CD19, CD20, CD45, CD13, CD33, y CD66. Analizamos la expresión de antígenos en función de los valores de intensidad media de fluorescencia (IMF) de las células B benignas maduras no leucémicas.

El análisis por citometría de flujo se llevó a cabo con un citómetro de flujo FACS Canto II (Becton Dickinson, Franklin Lakes, NJ) utilizando el software FACS Diva para la configuración del instrumento y la adquisición de muestras a lo largo del estudio. La adquisición de eventos para el panel de ocho colores en dos tubos incluyó todos los eventos mononucleares (identificados por su baja dispersión lateral en escala logarítmica frente a CD19), estableciéndose un recuento de detención superior a 500,000 eventos Se adquirió una cantidad mínima de 500.000 eventos en todas las muestras. El análisis de datos se realizó a partir de archivos en modo lista utilizando el software FACS DIVA, versión 8.0.2. Dos observadores independientes sometieron a todas las muestras a una estrategia unificada de *gating*, siguiendo un protocolo previamente establecido. La estrategia de análisis del panel de ocho colores consistía en incluir inicialmente todas las células B mediante una ventana citométrica (*gate*) basada en CD19 frente a las propiedades de baja dispersión lateral en escala logarítmica (SSC log), seguida de una caracterización adicional de las células B inmaduras según la expresión de los demás anticuerpos incluidos en el panel. La estrategia uniforme de ventanas utilizada en el ensayo de ocho colores consistía en el uso de ventanas parentales para aislar las células mononucleares y las células individuales (*singlets*), seguido de una ventana lógica basada inicialmente en la positividad para CD19/SSC log, con la subsiguiente definición de poblaciones de linfoblastos B y elementos hematógenos mediante ventanas adicionales basadas en CD19 frente a CD45 y CD19 frente a CD10. Se estableció que un resultado de enfermedad mínima residual (EMR) era positivo cuando se detectaba la presencia de una población de blastos aberrantes que representara al menos el 0,01 % del total de células mononucleares.

La identificación de la población de células B maduras benignas se realiza en función de las propiedades de dispersión lateral (SSC), expresión intensa de CD45, negatividad para CD10 y expresión brillante de CD20 dentro de las células seleccionadas mediante la ventana para CD19.

### EMR en las diferentes fases de la quimioterapia

Se recogieron los resultados de EMR para comparar los obtenidos en el momento del diagnóstico, día 0; en el día 35 tras la quimioterapia de inducción; en el día 52 tras la quimioterapia de consolidación; y durante la quimioterapia de mantenimiento en el día 78. Para analizar los cambios o la modulacion inmunofenotípica de los blastos, únicamente se seleccionó e incluyó en el estudio a aquellos pacientes con un resultado positivo de EMR en todas las fases.

Para la citometría de flujo diagnóstica, se emplearon muestras de sangre periférica o aspirado de médula ósea, mientras que el análisis de EMR se realizó exclusivamente en muestras de aspirado de médula ósea.

### Análisis estadístico

Utilizamos el programa SPSS versión 24.0 para calcular la frecuencia de género, la mediana y los rangos intercuartílicos (RIC) para la edad y los diferentes antígenos, esto es, CD10, CD45, CD20, TdT, CD34, CD19, CD66, CD13 y CD33. Empleamos la prueba de los rangos con signo de Wilcoxon para comparar la mediana de los valores de IMF en el momento del diagnóstico y en los momentos posteriores de EMR. La presencia de diferencias significativas en la expresión de antígenos se determinó mediante la prueba U de Mann-Whitney El valor de significancia se estableció en p<0,05.

## Resultados

Se incluyó un total de 203 casos de LLA-B con EMR persistentemente positiva durante el curso del tratamiento. La mediana de la edad del grupo fue de 5,7 años (RIC 3.0–8.0). En esta cohorte, 123 (60,6 %) eran niños y 80 (39,4 %) eran niñas. Analizamos la expresión de nueve antígenos en nuestro panel de población de blastos leucémicos de EMR de LLA-B, esto es: CD10, CD45, CD20, TdT, CD34, CD19, CD66, CD13 y CD33. Asimismo, evaluamos la expresión de tres antígenos en la población de linfocitos B benignos maduros: CD45, CD19 y CD20.

**Figura 1:**
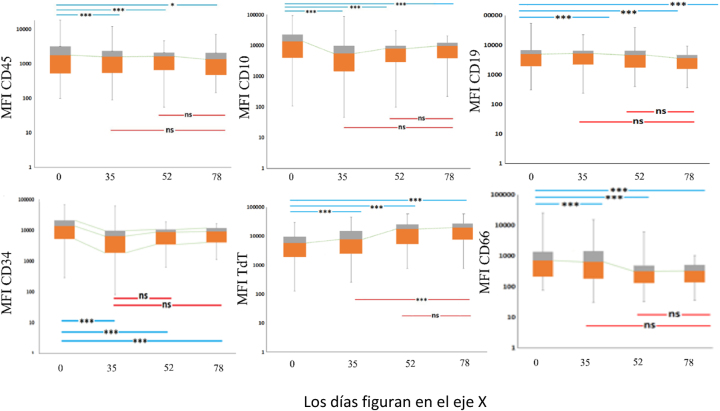
Modulación de las células leucémicas. Cambios en la IMF de CD10, CD45, CD34, CD66, CD19, y TdT durante la quimioterapia. D0, D35, D52 y D78. Los niveles de significación estadística se encuentran en cada caja sobre el intervalo correspondiente analizado (ns, no significativo; *p<0,05; ***p<0,01).

Observamos cambios estadísticamente significativos en los valores de IMF de seis de los antígenos expresados en los blastos, que incluyen, CD10, CD45, CD34, CD66, CD19, y TdT (Figura 1).

La caja abarca el intervalo intercuartílico (cuartiles inferior y superior) donde se concentra el 50 % de los datos, mientras que los bigotes (mínimo-máximo) resumen la distribución de los datos. Los valores medianos de las cohortes están representados por la línea discontinua, que refleja su evolución.

### Modulación de antígenos en los blastos

CD10: se produjo una modulación constante a la baja entre el día 0 y el día 35 (Z: −7,039; p<0,001); entre el día 0 y el día 52 (p<0,001); y entre el día 0 y el día 78 (p<0,001). Por el contrario, se produjo una modulación al alza no significativa entre el día 35 y el día 78 y entre el día 52 y el día 78.

CD45: así mismo, observamos una reducción significativa y constante de su expresión en la médula ósea en el día 35 (p=0,003), día 52 (p<0,001), y día 78 (Z: −2,181; p=0,029).

CD34: el análisis también mostró una disminución entre el día 0 y las fases posteriores, esto es, los días 35, 52 y 78 (p<0,001). Además, entre las fases de intervalo, dentro de cada fase se produjo una variación no significativa de la expresión.

CD66: identificamos una disminución constante significativa en los periodos 0–35 (p<0,001), 0–52 (p<0,001) y 0–78 (p<0,001). Advertimos una modulación a la baja no significativa en los periodos 35–78 y 52–78.

CD19: se produjo una disminución constante en los periodos 0–35 (p<0.001), 0–52 (p<0.001), y 0–78 (Z: −4.024; p<0.001).

TdT: Observamos un incremento estable en los periodos 0–35 (p<0,001), 0–52 (p<0,001), 0–78 (p<0,001) y 35–78 (p=0,021).

CD20: se detectó un aumento pequeño pero reversible en los periodos 0–35 y 0–78, y una disminución en el periodo 0–52. Sin embargo, el cambio no fue significativo en los otros tres periodos. En el periodo 35–78 se produjo una modulación significativa al alza (p=0,005).

CD33: el estudio mostró una tendencia variable en el antígeno CD33. Así, se observó un aumento estable pero no significativo en la expresión de CD33 en los periodos 0–35, 0–52 y 0–78, frente a una reducción estable entre las fases de intervalo en los periodos 35–78 y 52–78.

CD13: al igual que el antígeno CD33, la expresión de CD13 mostró tendencias variables entre fases, con un incremento reversible no significativo en el periodo 0–35, que fue reversible en las fases posteriores.

### Modulación de antígenos en células B benignas maduras

**Figura 2:**
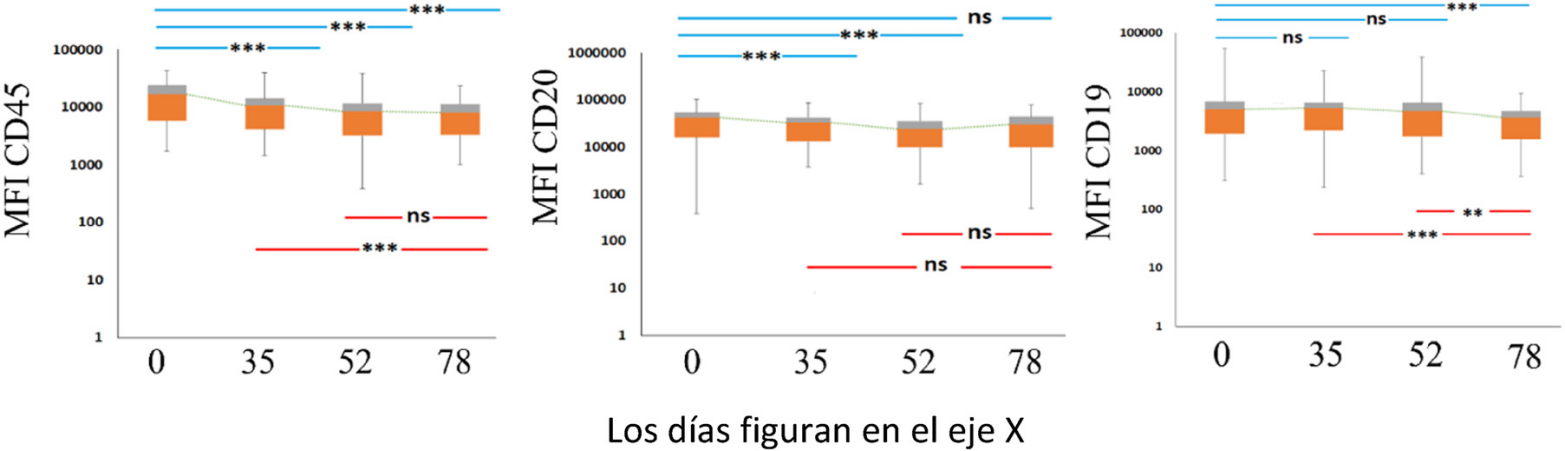
Modulación de los linfocitos B residuals benignos. Cambios en la IMF de CD45, CD20, y CD19 durante la quimioterapia. D0, D35, D52 y D78. Los niveles de significación estadística figuran en cada caja encima del interval correspondiente analizado (ns, no significativo; *p<0,05; ***p<0,01).

Con el fin de investigar las variaciones en la expresión de antígenos y evaluar el efecto de la terapia en la expresión de antígenos en los linfocitos B benignos maduros en la muestra, analizamos las tendencias en la expresión de CD45, CD20, y CD19 entre los día 0, 35, 52 y 78, respectivamente. En la población de linfocitos B benignos maduros (Figura 2), la expresión de CD45 se redujo de manera constante y significativa en los periodos 0–35 (p<0,001); 0–52 (p<0,001); 0–78 (p<0,001) y 35–78 (p=0,003). Del mismo modo, la expresión de CD20 fue disminuyendo de manera constante y significativa entre los intervalos 0–35 (p<0,001) y 0–52 (p<0,001). Con respecto a la expresión de CD19, esta se redujo de manera constante pero no significativa en los periodos 0–35 y 0–52, disminuyendo de manera significativa en los periodos 0–78 (p<0,001), 35–78 (p=0,003) y 52–78 (p=0,017).

La caja abarca el intervalo intercuartílico (cuartiles inferior y superior) donde se concentra el 50 % de los datos, mientras que los bigotes (mínimo-máximo) resumen la distribución de los datos. La línea discontinua representa la evolución de los valores medianos de las cohortes.

### Análisis comparativo de los linfocitos B benignos maduros y las células leucémicas

También comparamos la expresión de los antígenos CD45, CD20, y CD19 en las células leucémicas frente a las células benignas, evidenciando una diferencia notable en el momento del diagnóstico y, consistentemente, en las diferentes fases de la quimioterapia, tal como se puede observar en la Tabla 1.

**Tabla 1:** Comparación de la MI en la población de linfocitos B residuals y la población de células blásticas leucémicas mediante la prueba de Mann-Whitney test.

Mediana (IQR)	Día 0	Valor p	Día 35	Valor p	Día 52	Valor p	Día 78	Valor p
CD45-L	1.266 (532,8–2.700)	0,000^a^	1.060,8 (550,9–1.814,7)	0,000^a^	997,5 (661,3–1.466,9)	0,000^a^	889 (479,1–1.588,3)	0,000^a^
CD45-B	10.675 (5.964–17.906)		6.544 (4.239–9.842)		5.395 (3.213–8.409)		4.622 (3.340–8.041)	
CD20-L	10.71,6 (360–3.591,6)	0,000^a^	12.33,5 (506,2–27.81,5)	0,000^a^	935 (524,4–2.165)	0,000^a^	13.89,5 (705,5–3.260,7)	0,000^a^
CD20-B	26.091 (16.393–37.556)		20.112 (13.229–30.325)		14.696 (10.011–26.219,5)		20.085 (9.977–34.322)	
CD19-L	2.376 (1.533–4.571)	0,007^a^	1.968 (1.206–3.309)	0,000^a^	14.74,2 (10.47,7–20.09,2)	0,000^a^	15.01,3 (10.37,3– 22.31,5)	0,001^a^
CD19-B	3.079 (1.919–4.846)		3.012 (2.219–4.299)		2.890 (1.754–4.717)		2.142 (1.572–3.120)	

^a^Nivel de significación p; L, población de células blásticas leucémicas; B, población cellular benigna (linfocitos b residuales); MI, modulación inmunofenotípica.

## Discusión

Se ha demostrado que la determinación de la EMR posee un importante valor pronóstico en la LLA. Tras la quimioterapia, con frecuencia se producen alteraciones inmunofenotípicas en las células leucémicas, debido principalmente al tratamiento con glucocorticoides (GC). El presente estudio revela cambios estadísticamente significativos en los valores de IMF de seis antígenos de diferenciación (CD) expresados en los blastos leucémicos, que son: CD10, CD45, CD34, CD66, CD19, y TdT, a diferencia de los antígenos CD20, CD13, y CD33, en los que no se observaron cambios significativos.

Dicho hallazgo debe ser motivo de atención, ya que la expresión de marcadores importantes como CD34, CD10, CD19, y CD45 puede variar, afectando con ello a la detección de EMR. Los cambios pueden también afectar a la intensidad de la expresión, la pérdida o adquisición de antígenos, o a cambios de linaje [9]. Un estudio reveló que existe una probabilidad aproximada del 25 % de no detectar EMR en los marcadores primarios [10]. La modulación de antígenos en las células leucémicas también se ha documentado en otros estudios en los que se aplicó el protocolo de inducción de Berlin-Frankfurt-Munich [6], [7], [11], lo que coincide con los resultados de nuestro estudio.

Hasta el día 78, se produjo una disminución constante del antígeno CD10 con respecto al momento del diagnóstico, algo que Burnusuzov et al. también observaron [6]. El otro estudio realizado por Dworzak et al. mostró una disminución significativa en la expresión de CD10 hasta el día 35, para recuperarse posteriormente hasta el día 78 a valores comparables a los del momento del diagnóstico [7]. Se produjo una disminución reversible de la expresión del antígeno CD34 en nuestra cohorte, habiendo mostrado tendencia a aumentar en las fases posteriores, aunque sin alcanzar el límite de los niveles del momento del diagnóstico. En la misma línea, Dworzak et al. observaron una modulación reversible, especialmente en la expresión de CD34, que se mostró notablemente variable durante el seguimiento. Además, en algunos pacientes, su expresión tendió a aumentar en la fase de post inducción tras la reducción gradual de la dosis de glucocorticoides. Este patrón reversible se podría atribuir a la quimioterapia con glucocorticoides, coincidiendo con el régimen de glucocorticoides [12].

Otros estudios también mostraron diferencias en la expresión documentada de CD34 durante la quimioterapia. Gaipa et al. [13] observaron una reducción de la expresión de CD23, lo que contrasta con los resultados de Thulasi et al. [14] y con Cap et al. [15], que observaron un aumento de la expresión de CD34 tras la reducción gradual de GC. Los resultados del presente estudio muestran que se produce una modulación reversible y significativa en la expresión del antígeno CD34, lo que contribuye a mejorar nuestra comprensión de su comportamiento durante la quimioterapia.

En contraposición a lo descrito por Dworzek et al., nuestro estudio reveló una disminución constante y significativa de la expresión de CD45 durante el periodo transcurrido entre el día 0 y el día 78 [7]. Por otro lado, Gaipa et al. no observaron alteraciones significativas en la expresión de CD45 en ninguno de los periodos de observación [4]. En nuestro estudio, detectamos que la expresión de CD66 se mantenía estable, experimentando una reducción significativa a lo largo de las diferentes fases de la quimioterapia. El estudio llevado a cabo por Ismail et al. también mostró una disminución de la expresión del antígeno CD66 [16].

En la literatura, se ha documentado una modulación al alza del antígeno CD19 en las primeras semanas de quimioterapia hasta el día 15, seguidas de una disminución notable en las fases posteriores, lo que coincide con los hallazgos de nuestro estudio. Así, identificamos una reducción constate significativa entre el día 0 y el día 78, con una disminución no significativa en los días 35 a 78. Estos resultados coinciden con la disminución documentada por Chowdhury et al. en la expresión del antígeno CD19 tras una semana de quimioterapia [17]. En nuestro estudio, observamos una modulación al alza estable y significativa en la expresión de TdT (tras la prednisona), lo que contrasta con los resultados obtenidos por Akiyama et al., que hallaron una reducción en la expresión del gen TdT en la LLA-B inducida por la prednisona [18].

Así mismo, detectamos una modulación reversible no significativa del marcador CD20. Burnusuzov et al. comunicaron un pequeño aumento no significativo del antígeno CD20 [6]. El otro estudio reveló un aumento el marcador CD20 [8].La expresión y persistencia de los antígenos mieloides CD13 y CD33 mostraron una notable variabilidad.

Se observó un aumento reversible en la expresión de CD13. Entre las 168 muestras con resultado positivo para la expresión de CD13 en el día 0, se observaron resultados reproducibles en 17 casos (10 %) que siguieron siendo positivos en los días 35, mientras que 7 casos (4 %) fueron positivos el día 52. En el caso de CD33, identificamos un incremento estasble entre el día 9 y el día 78. De nuevo, del total de 173 casos positivos para CD33 en el día 0, observamos positividad reproducible y persistente en 10 casos (5.7 %) en el día 35, 8 casos (4,6 %) en el día 52, y 5 casos (28 %) en el día 78.

En nuestra investigación, evaluamos el impacto de la IM únicamente en la población leucémica mediante el análisis de variaciones en la expresión de CD19, CD20, y CD45 en linfocitos B benignos maduros. En estudios anteriores, se había demostrado la naturaleza regulada de los patrones de expresión de antígenos en las células B maduras normales (linfocitos). En un estudio, se observó una disminución estadísticamente significativa del antígeno CD19 entre los días 15 y 33 (p=0,008) en la expresión de CD19 en células benignas [11]. Sin embargo, nuestro estudio revela una reducción no significativa pero constante entre el día 0 y el día 35.

Además, los resultados publicados indican un incremento mayor de la expresión de CD20 y CD45 en los linfocitos B que en las células leucémicas [11]. En nuestro estudio, observamos una modulación reversible en las células leucémicas y una reducción constante de CD20 en las células benignas. Así mismo, se detectó una disminución del antígeno CD45 tanto en las poblaciones de células leucémicas como benignas. La identificación detallada de patrones de expresión de antígenos permite realizar una evaluación más completa de los efectos inmunodulatorios tanto sobre las poblaciones de células B normales como leucémicas.

Durante la fase inicial de la terapia de inducción, resulta particularmente difícil diferenciar y detectar los blastos leucémicos, debido a las alteraciones inmunofenotípicas inducidas por los agentes antineoplásicos, la coexistencia de hematógonos, y la ausencia de un inmunofenotipo asociado a la leucemia (IAL). El uso de la citometría de flujo de ocho colores, sumado al empleo de un amplio panel de anticuerpos y un conocimiento detallado de las tendencias de cambio inmunofenotípico inducidas por la terapia, permite predecir y estudiar la enfermedad a través del análisis de la modulación de diversos fenotipos inmunológicos, tanto en linfocitos B malignos como normales. Con el empleo de otros marcadores de diferenciación (CD) estables en el panel de EMR, se puede diferenciar de manera inequívoca la población de EMR en todos los tipos de muestras.

La estrategia de *gating* en la citometría de flujo para medir la EMR en la leucemia linfoblástica aguda es susceptible de cierta subjetividad y variabilidad durante el proceso de *gating*. Cada investigador puede definir las ventanas citométricas (*gates*) de manera distinta, pudiendo así dar lugar a inconsistencias en los resultados. La presencia de poblaciones superpuestas, así como niveles bajos de enfermedad residual pueden complicar la identificación y cuantificación precisa de las células diana. Sin embargo, en nuestro estudio, se aplicó a todas las muestras una estrategia unificada de *gating* siguiendo todos los técnicos el mismo protocolo de *gating*, definido con claridad en los procedimientos operativos normalizados. Dos observadores independientes analizaron cada caso meticulosamente, lo que permitió reducir notablemente la variabilidad en el análisis y la interpretación de los resultados.

### Limitaciones

El presente estudio se centra exclusivamente en la modulación de antígenos en casos de LLA pediátrica, habiendo quedado excluidos los casos de LLA en adultos, lo que supone una limitación. Es perentorio seguir investigando diferentes combinaciones de marcadores, incluyendo los descubiertos recientemente, con el fin de mejorar la sensibilidad y especificidad de la prueba.

## Conclusiones

En conclusión, este estudio aporta pruebas contundentes de que los pacientes pediátricos con LLA-B sometidos a quimioterapia presentan una modulación inmunofenotípica significativa. Las fluctuaciones observadas en la IMF en diferentes antígenos clave como CD10, CD19, CD34, CD45, TdT, y CD66 evidencia la naturaleza dinámica de los blastos leucémicos durante el tratamiento. Aunque la detección de EMR sigue siendo la piedra angular del manejo de la enfermedad, la modulación de antígenos inducida por la quimioterapia hace preciso realizar un análisis cauteloso de la EMR. Si ignoramos dichos cambios, corremos el riesgo de realizar una evaluación imprecisa de la EMR, lo que podría afectar a la estratificación del riesgo del paciente y a las estrategias de tratamiento. De este modo, realizar un análisis meticuloso de los cambios inmunofenotípicos resulta crucial para garantizar la fiabilidad de la detección de EMR. Es necesario realizar más estudios para seguir desgranando los mecanismos que producen dichas modulaciones, así como desarrollar protocolos normalizados para mitigar su impacto en la interpretación de la EMR.
